# Molecular Mechanisms by Which Imeglimin Improves Glucose Homeostasis

**DOI:** 10.1155/2020/8768954

**Published:** 2020-03-06

**Authors:** Habib Yaribeygi, Mina Maleki, Thozhukat Sathyapalan, Tannaz Jamialahmadi, Amirhossein Sahebkar

**Affiliations:** ^1^Research Center of Physiology, Semnan University of Medical Sciences, Semnan, Iran; ^2^Chronic Kidney Disease Research Center, Shahid Beheshti University of Medical Sciences, Tehran, Iran; ^3^Academic Diabetes, Endocrinology and Metabolism, Hull York Medical School, University of Hull, Tehran, Iran; ^4^Biotechnology Research Center, Pharmaceutical Technology Institute, Mashhad University of Medical Sciences, Mashhad, Iran; ^5^Department of Nutrition, Faculty of Medicine, Mashhad University of Medical Sciences, Mashhad, Iran; ^6^Halal Research Center of IRI, FDA, Tehran, Iran; ^7^Neurogenic Inflammation Research Center, Mashhad University of Medical Sciences, Mashhad, Iran; ^8^School of Pharmacy, Mashhad University of Medical Sciences, Mashhad, Iran

## Abstract

Despite different classes of antidiabetic medications available for the management of patients with diabetes, efforts are underway to identify novel and safer antihyperglycemic agents with higher potency and increased tolerability. Imeglimin is a promising antidiabetic agent that has shown to have significant antihyperglycemic effects in studies, although it has not been approved yet. There is growing evidence that imeglimin improves glucose homeostasis in the diabetic milieu; however, the precise molecular mechanisms are still not elucidated. In this review, we discuss various molecular pathways by which imeglimin exerts its antihyperglycemic effects and improves glucose homeostasis in the diabetic milieu.

## 1. Introduction

The prevalence of diabetes mellitus (DM) is growing rapidly worldwide, especially among younger adults [1]. DM and its complications contribute to significant morbidity and mortality globally [1, 2]. DM has a negative effect on most metabolic pathways, including oxidative stress, inflammation, apoptosis, and necrosis leading to the development of various complications associated with diabetes [3, 4]. Diabetes and its associated complications have a substantial economic burden on health systems in most countries [5, 6]. Many pharmacological agents have been developed to improve hyperglycemia and prevent complications associated with diabetes [7]. Imeglimin is a novel and promising antihyperglycemic agent but has not been approved yet for managing patients with diabetes [8, 9]. All of the pharmacological properties of imeglimin and the mechanisms behind its therapeutic effect have not been fully elucidated [8, 10]. We searched for related articles using keywords of imeglimin and diabetes mellitus in various databases such as PubMed, Medline, and Scopus which evaluated the possible mechanism of action of imeglimin in diabetes. Based on this, in the current review, we discuss the possible molecular pathways by which imeglimin improves glucose homeostasis in the diabetic milieu.

## 2. Imeglimin

Imeglimin, with the chemical name of (6R)-(+)-4-dimethylamino-2-imino-6-methyl-1,2,5,6-tetrahydro-1,3,5-triazine hydrochloride, is a new promising antidiabetic medication which has demonstrated antihyperglycemic effects in various studies [8, 11]. It is an inhibitor of the oxidative-phosphorylation process taking place inside the mitochondria of aerobic cells and thereby can exert potent metabolic effects in eukaryotic cells [12]. Imeglimin is the first member of oral tetrahydrotriazine-containing chemical compounds, the glimins, with promising antidiabetic effects. Imeglimin has recently completed its phase 2b and currently is in phase 3 trial in Japan [13–15]. It is primarily developed as an add-on treatment for combination therapy with other agents to improving insulin secretion and sensitivity in patients with type 2 diabetes (T2DM) [14, 16, 17]. Further investigations have demonstrated that it might provide metabolic effects by improving glucose and lipid homeostasis in the diabetic milieu [11, 18, 19]. Positive reports from phase 2b trials indicated that it could reduce glycosylated hemoglobin (HbA1c) as monotherapy in a dose-dependent manner with a favorable tolerability and safety profile in patients with T2DM [13, 20]. Moreover, it has suggested that imeglimin corrects three fundamental defects commonly observed in patients with T2DM, including a higher rate of gluconeogenesis, low glucose-induced insulin secretion from beta cells, and peripheral insulin resistance [9, 19]. Therefore, it has potential advantages to other oral hypoglycemic agents, which target only one or two defects and not all the three defects, namely, increased glycogenesis in the liver, impaired insulin secretion from the pancreas, and insulin resistance in muscles. Thereby, imeglimin is one of the potentially promising medications for managing patients with T2DM, if approved [8] (Table 1).

## 3. Antidiabetic Potentials of Imeglimin

**Figure 1:**
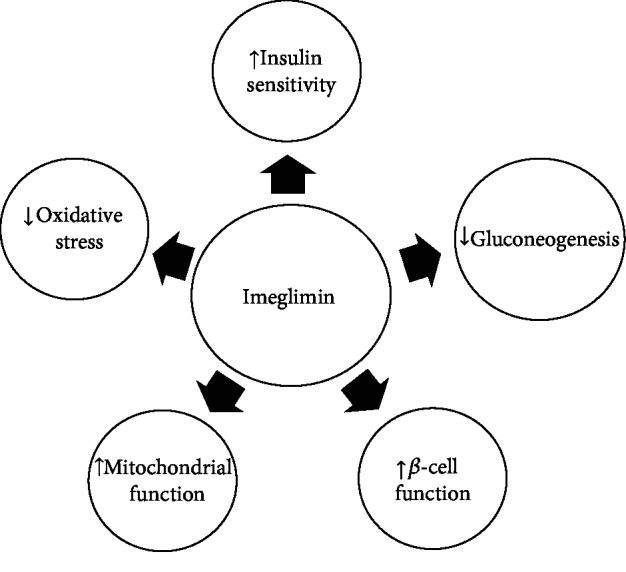
Possible molecular mechanisms by which imeglimin improves glucose homeostasis.

Emerging in vitro and in vivo evidence suggests that imeglimin has potent antihyperglycemic effects and is able to normalize glucose homeostasis through several pathways [8–10, 18]. In the following sections, we review the possible molecular mechanisms by which imeglimin exerts its pharmacological effects (Figure 1).

### 3.1. Imeglimin and Insulin Sensitivity

Insulin resistance in peripheral tissues is a central feature of T2DM as well as gestational diabetes, which inhibits glucose entering into the insulin-dependent cells as adipocytes, skeletal myocytes, and cardiomyocytes [21]. Imeglimin can induce insulin sensitivity through several molecular pathways [18]. It can promote insulin signal transduction by increasing Akt (protein kinase B) phosphorylation [18]. Vial and colleagues in 2015 showed that imeglimin increased insulin sensitivity in high-fat diet mice [18]. Also, Pacini and coworkers in 2015 demonstrated that imeglimin induces insulin sensitivity in the beta-cells of patients with T2DM [9]. They suggested that imeglimin can increase peripheral insulin sensitivity in the diabetic milieu [9]. Although the underlying mechanisms of these insulin-sensitizing effects of imeglimin are not clearly understood, this might potentially include glucose transporter-4 (Glut-4) expression and modulating insulin receptor substrate (IRS) phosphorylation.

### 3.2. Imeglimin and Gluconeogenesis

Hepatic gluconeogenesis is a physiologic process in hepatic cells in which they produce glucose using other substrates; however, excess hepatic gluconeogenesis occurs in the diabetic milieu [22]. Imeglimin has been used to reduce hepatic gluconeogenesis [8, 19]. Fouqueray and coworkers in 2011 demonstrated that imeglimin markedly reduced the gluconeogenesis by downregulating the phosphoenolpyruvate carboxykinase (PEPCK) and glucose-6-phosphatase (G6Pase) in isolated hepatocytes from rats [19]. Wagner et al. in 2012 showed that imeglimin improved glucose homeostasis by modulating hepatic gluconeogenesis in diabetic mice [12]. Moreover, Vial and colleagues in 2014 demonstrated that imeglimin reduced the hepatic gluconeogenesis by inhibition of lactic acidosis via the mitochondrial-dependent pathway [23]. This evidence demonstrates that the suppression of hepatic gluconeogenesis by imeglimin helps to reduce the level of circulatory glucose and attenuates hyperglycemia.

### 3.3. Imeglimin, *β*-Cell Function, and Insulin Secretion

Evidence suggests that imeglimin preserves beta-cell function and increases glucose-induced insulin secretion [11]. Perry et al. in 2016 found that imeglimin corrects glucose homeostasis and reduces HbA1c by directly stimulating insulin secretion and improving the function of pancreatic islets in diabetic mice [11]. Hallakou-Bozec et al. in 2016 demonstrated that imeglimin stimulates postprandial insulin secretion by a nicotinamide adenine dinucleotide- (NAD-) dependent mechanism and activating salvage pathway in a dose-dependent manner in islets of diabetic rats [24]. Pacini and coworkers in 2015 demonstrated that imeglimin protected beta-cells and promoted islet function by improving glucose homeostasis in the diabetic milieu [9]. Lablanche and colleagues in 2018 provided further data indicating imeglimin attenuated beta-cell apoptosis by lowering the glucotoxicity by a mitochondrial-dependent mechanism [25]. They also suggested that imeglimin increases beta cell mass by inhibitory impacts on permeability transition pores (PTP) of mitochondria [25]. There is a growing evidence confirming the protecting roles of imeglimin on beta cells [9, 26]. The above mentioned evidence suggests that imeglimin improves glucose homeostasis partly via promoting beta-cell function.

### 3.4. Imeglimin and Mitochondrial Function

Mitochondrial dysfunction is common in DM, which impairs the insulin-dependent cells (adipocytes, cardiomyocytes, and myocytes) to an adequate response to circulatory insulin [27]. It also has a negative effect on pancreatic beta-cells and reduces the production and release of insulin in response to circulatory glucose [27, 28]. Thus, preserving the mitochondrial function is an important aspect in the management of diabetes [27]. Some evidence suggests that imeglimin can improve mitochondrial function in patients with diabetes [18, 29]. Vial and coworkers in 2015 demonstrated that imeglimin improved mitochondrial function by modulating complexes I and III activities, promoting mitochondrial fatty acid oxidation and by normalizing phospholipid composition in the mitochondria of diabetic animals [18] which resulted in improved glucose homeostasis in these animals [18]. Detaille et al. in 2016 demonstrated that imeglimin regulated mitochondrial PTP and preserved mitochondrial function in cultured human endothelial cells [13].

### 3.5. Imeglimin and Oxidative Stress

Oxidative stress, which refers to the imbalance between free radical species and antioxidative system in the biologic milieu, has an important role in the pathogenesis of DM and its complications [21, 30]. It can markedly impair various insulin signaling pathways and induces insulin resistance [30]. Recent evidence indicates that imeglimin has antioxidative potentials which enables it to ameliorate free radical generation and readjust the redox state [18]. For example, Vial and colleagues in 2015 reported that imeglimin attenuated oxidative stress by suppressing the mitochondrial free radical generation leading to improved glucose homeostasis [18]. Detaille and coworkers in 2016 found that imeglimin reduced mitochondrial free radicals in cultured human endothelial cells [13]. Lablanche et al. in 2018 reported that imeglimin ameliorated hyperglycemia-induced oxidative damages in rat insulinoma cell line INS-1 [25]. The above studies demonstrate the antioxidative potentials of imeglimin.

## 4. Other Beneficial Effects of Imeglimin in DM and Its Complications

Beyond the above described metabolic effects, imeglimin can provide additional pharmacologic effects [26]. For example, it improves endoplasmic reticulum stress and in turn inhibits various downstream pathophysiologic pathways [26]. It could also potentially act as a protective agent against vascular dysfunction in diabetic complications [13, 31]. There is some evidence suggesting that imeglimin improves endothelial dysfunction and vascular network, thereby could be a new therapeutic agent for various diabetes-induced vascular disorders such as nephropathy and retinopathy [13, 31, 32]. However, more experimental clinical studies are still required to demonstrate this effect.

### 4.1. Clinical Evidence

In addition to experimental studies, there is clinical evidence demonstrating the antidiabetic properties of imeglimin in human [9, 14]. It should be noted that there are some differences between the findings of experimental studies and clinical trials since clinical studies are associated with some variability in effectiveness and some adverse effects which may not have occurred in experimental studies. In experimental studies, we can precisely survey the involved molecular mechanisms and control most of the confounding factors which may not be controlled in the real environment in clinical trials. Besides, drug dosing, compliance to the assigned treatment, and continuous monitoring cannot be achieved in the clinical setting with the same precision as experimental studies. Therefore, clinical trials usually have lower efficiency compared to experimental studies.

Pacini and coworkers in 2015 demonstrated that after one week of imeglimin therapy (1500 mg/12 h), there was an improvement in beta-cell function in patients with T2DM [9]. Pirags and colleagues in 2012 reported that imeglimin could be as effective as metformin in improving the glucose homeostasis in patients with T2DM [14]. Fouqueray and coworkers in 2013 found that imeglimin can improve glycemic control in patients with T2DM who are not adequately controlled with metformin [16]. More related clinical evidence is presented in Table 2.

## 5. Conclusion

Imeglimin is the first member of promising oral antidiabetic agents known as glimins, which are currently is in phase 3 trial. It is an inhibitor of the oxidative-phosphorylation process and thereby can provide potent metabolic effects, including on glucose homeostasis. There is growing evidence that imeglimin reduces postprandial hyperglycemia, normalizes glycated hemoglobin, and improves beta-cell function in patients with T2DM. Although more clinical trials are required to elucidate the exact molecular effects of this medication, available evidence suggests that imeglimin improves glucose homeostasis via five different molecular pathways including lowering insulin resistance, suppressing the gluconeogenesis, improving beta-cell function, improving mitochondrial function, and attenuating the oxidative stress. Although these molecular mechanisms have complex interactions with each other, and it may be impossible to consider them as separate pathways, based on current knowledge, all these pathways are heavily involved in the antihyperglycemic effects of imeglimin. In the current study, the endpoint was the insulin-sensitizing capabilities of imeglimin; however, it may exert other beneficial impacts such as renoprotective and cardioprotective which needs to be evaluated in future studies. Moreover, other possible molecular pathways may be involved which have not been evaluated yet. For example, modulatory effects of imeglimin on inflammatory responses, possible effects on adipokines and adiponectins, and the possible effects of imeglimin on glucagon secretion as well as other molecular pathways by which imeglimin induces insulin sensitivity could be examined in future studies to recognize all aspects of the pharmacologic potentials of imeglimin.

## Figures and Tables

**Table 1:** Possible molecular mechanisms by which imeglimin improves glucose homeostasis.

Mechanisms	Effects	Ref.
Insulin sensitivity	Increases insulin sensitivity and reduces insulin resistance via different molecular pathways such as promoting Akt phosphorylation	[9, 18]
Gluconeogenesis	Modulates genes involved in hepatic gluconeogenesis as PEPCK and G6Pase, declined gluconeogenesis	[12, 19, 23]
*β*-Cells' function and insulin secretion	Protects against beta-cell death, increases beta cell mass, and improves glucose-induced insulin release from islets	[9, 11, 24–26]
Mitochondrial function	Improves mitochondrial function in beta cells as well as other tissues	[13, 18, 29]
Oxidative stress	Reduces mitochondrial-induced free radical generation, declines hyperglycemia-dependent oxidative stress, and in turn ameliorates oxidative damages	[13, 18, 25]

Akt = protein kinase B; PEPCK = phosphoenolpyruvate carboxykinase; G6Pase = glucose-6-phosphatase.

**Table 2:** Main clinical evidences about antihyperglycemic effects of imeglimin.

Study population	Dosage	Duration of study	Effects	Ref.
33 patients with T2DM	1500 mg/12 h	7 days	Improved beta cell function, increased postprandial insulin release	[9]
59 patients with T2DM	2000 mg/day	4 weeks	Was as effective as metformin in reducing HbA1c, has more safety and tolerability than metformin	[14]
156 patients with T2DM	3000 mg/day	12 weeks	Decreased HbA1c, improved glycemic control	[16]
170 patients with T2DM	3000 mg/day	12 weeks	Reduced HbA1c and FBS, showed more safety than sitagliptin monotherapy	[17]
73 T2DM patients	1000-3000 mg/day	24 weeks	Improved plasma glucose control, showed good efficacy and safety especially in a dose of 2000 mg/day	[20]
